# Barriers to and facilitators of pet grooming among clients served by a subsidized grooming service program

**DOI:** 10.3389/fvets.2022.1021707

**Published:** 2022-10-12

**Authors:** Shelby E. McDonald, Colleen Doherty, Jessica Sweeney, Lisa Kisiel, Angela Matijczak, Laura Niestat, Maya Gupta

**Affiliations:** ^1^Department of Strategy and Research, American Society for the Prevention of Cruelty to Animals, New York, NY, United States; ^2^Community Engagement Program, Department of Humane Law Enforcement, American Society for the Prevention of Cruelty to Animals, New York, NY, United States; ^3^School of Social Work, Virginia Commonwealth University, Richmond, VA, United States; ^4^Veterinary Forensic Sciences, Department of Humane Law Enforcement, American Society for the Prevention of Cruelty to Animals, New York, NY, United States

**Keywords:** grooming, pets, hygiene, cruelty prevention, spectrum of care, access to veterinary care, community engagement

## Abstract

Grooming is an important aspect of basic hygiene care for most companion animals. The consequences of not receiving routine grooming care can pose significant risks to animals' health and wellbeing. The current study examined barriers and facilitators of maintaining pets' grooming needs among clients of a subsidized grooming service program in New York City (*N* = 167), as well as the impact of a tailored nail-trimming demonstration on clients' confidence trimming pets' nails. Ninety-two percent of the sample reported experiencing at least one barrier to maintaining their pet's grooming (e.g., income, transportation) and nearly half (46%) experienced three or more barriers to providing grooming. Ninety-one percent endorsed that at least one supply/support (e.g., brush/comb, behavioral support) would be beneficial in maintaining their pet's grooming needs at home and more than half reported that three or more supplies/services would be beneficial. Differences in the prevalence of specific barriers to grooming were found across income groups, service locations, and service settings. Clients who received nail-trimming demonstrations, on average, reported statistically significant increases in confidence trimming nails following their appointment. We discuss the implications of these findings for improving animal welfare and veterinary professionals' capacity for preventing grooming-related omissions of care and increasing communities' capacity to support pet owners' access to essential pet care supplies and supports. Future research is needed to determine (a) how and for whom grooming demonstrations and subsidized services are most effective, (b) whether an increase in pet owner confidence following nail trimming demonstrations is associated with maintaining nail trimming at home over time, and (c) whether providing clients with supplies and supports is an effective way of preventing and/or ameliorating future grooming-related omissions of care and hygiene-related health concerns observed by veterinarians, animal control professionals, shelter staff, and law enforcement.

## Introduction

Grooming is an important aspect of basic hygiene care for most companion animals and can include activities such as bathing, trimming nails, cleaning ears, and brushing or clipping hair. Grooming may also include flea and tick baths and dips that provide prevention and treatment of parasites. The potential health consequences of inadequate grooming are diverse and can range in severity. For example, the consequences of matted hair can range from skin irritation and discomfort to constricted blood flow and lymphatic drainage, resulting in soft tissue death and bone injury (1). Nails that are not trimmed may affect the anatomic position and function of the feet and alter an animal's normal gait (1, 2). In severe cases, nails may grow in a circular pattern and cause painful wounds as they penetrate the paw pads (1–3). Thus, the consequences of grooming-related omissions of care have serious implications for animal health and welfare (1–4).

Studies examining pet caregivers' grooming practices are limited. An important step in preventing grooming-related omissions of care is to understand the reasons that caregivers may not provide adequate grooming (4). A 2020 survey conducted by the American Pet Products Association found that most canine caregivers groom their dogs at home (41%) or take their pets to a full-service salon (30%), with a smaller number of people indicating the use of mobile grooming services^1^ (9%), retailers (8%), or a self-service center (6%) (5). On average, canine caregivers reported that their dogs were groomed professionally about four times in the past year (5). A 2022 report on pet ownership during the COVID-19 pandemic found that between 40 and 49% of U.S. pet caregivers used one of three types of grooming services (mobile, full-service, and/or in-retail services) (6). Among those who used grooming services, between 10 and 16% indicated their use of these services had changed as a result of the COVID-19 pandemic, with nearly two-thirds of these pet owners indicating that their use of these services had decreased. Cost was the most frequently reported reason for decreasing service use (31–41%), followed by the lack of available services (15–22%) (6).

The American Society for the Prevention of Cruelty to Animals (ASPCA) recently reported on the scope of grooming-related concerns among animals served by ASPCA programs in New York City. Specifically, McDonald et al. (4) retrospectively extracted program data (e.g., appointment notes, appointments with medically necessary grooming or nail trim) from five ASPCA programs and found that the prevalence of grooming-related concerns among animals served was relatively consistent across three of the ASPCA's veterinary service programs (4–6% of appointments). This study also identified that 13% of the ASPCA-NYPD Partnership's cruelty cases involved general hair matting concerns and/or strangulating hair mat wounds, of which 93% involved long-haired dog breed types. Collectively, results of this study indicated that improving access to grooming services, facilitating access to grooming supplies, and improving caregivers' knowledge of their pet's grooming needs may be likely to improve the welfare of a significant number of companion animals served by partially or fully subsidized animal welfare program services (4). To develop and implement effective programs, it is important that animal welfare and veterinary professionals understand what factors are related to pet owners' access to grooming services and supplies, awareness of their pet's grooming needs, and their ability to groom their pet(s) (4). Several potential barriers to maintaining grooming have been hypothesized, including lack of available veterinary, grooming, and pet supply resources within a reasonable distance of one's home, lack of pet-friendly or reliable transportation, low financial resources, owners' beliefs about health-promoting behaviors, and animal stress and behavioral problems (4, 7–9).

### Current study

The current study, which seeks to advance knowledge in this area by reporting on data collected as part of a pilot study of a subsidized grooming services program in New York City, has four aims. Our first aim was to better understand how dog and cat owners served by the ASPCA's grooming services program view the role of grooming in relation to maintaining their pet's health and their confidence performing basic components of pet grooming (e.g., bathing, brushing hair, trimming nails). Although this aim was primarily exploratory, we anticipated that pet owners would be least confident trimming their pet's nails. Our second aim was to identify factors that may serve as barriers to pet owners' ability to provide basic grooming care. We hypothesized that dog and cat owners served by the program would, on average, experience multiple barriers to grooming-related care, including finances, transportation, physical ability, time, pets' behavior, and proximity to grooming services and supply stores. In addition, we anticipated that income would be the most prevalent barrier to grooming pets. Our third aim was to identify what supplies and supports are needed for dog and cat owners to maintain their pet's grooming needs at home. We hypothesized that most pet owners would benefit from varied supplies and support services based on their unique needs, including basic grooming supplies (e.g., brushes, nail clippers) and behavioral support services for pets. Our final aim was to test whether nail trimming demonstrations provided through the program significantly improved pet owners' confidence cutting their pet's nails. We hypothesized that, on average, pet owners' confidence trimming nails would increase following the demonstration.

## Methods

### ASPCA subsidized grooming program

The ASPCA's Community Engagement (CE) team works with New York City residents who lack access to vital veterinary care, services, and supplies for their pets. Often, these pet owners are referred to the team by the New York City Police Department (NYPD) following a response to animal welfare/cruelty complaints, as cases deemed more appropriate for supportive services than criminal justice system involvement. The CE team provides families with resources to help them create and sustain a safe and healthy environment for their pet(s). The team also accepts client referrals from social service and other allied agencies and conducts outreach throughout the community to increase awareness and access to the ASPCA's veterinary and spay/neuter services. A grooming program is provided at the ASPCA's stationary Community Veterinary Clinics (CVCs) located in the Bronx and Brooklyn, in addition to mobile grooming events throughout the city. Program staff engage in targeted service promotion by distributing flyers locally, partnering with agencies such as the NYPD's Community Affairs Unit, and relying on word-of-mouth to reach prospective clients.

Typically, grooming services address basic needs such as nail trimming and sanitary cuts (shave of the belly and rear area of the dog to keep this area more sanitary), which can be done quickly and efficiently. Full-service medical grooming (e.g., full body shave-down or sedated grooming, which involves veterinary supervision) is also available at CVC locations. Nail trimming demonstrations were provided by the groomer using the participant's own pet during which the groomer performed basic aspects of nail trimming (e.g., holding paws, cutting nails, filing nails, use of styptic powder) and provided verbal instructions. Client eligibility includes residency of the borough in which the services are taking place and that clients report $50,000 or less in total household income. Pet owners do not need to be an existing ASPCA client to get an appointment, and the pet does not need to be vaccinated (however, the ASPCA prioritizes providing vaccines either on the same day or at a future appointment). The type of appointment a client receives (e.g., nail trim demonstration, sanitary cut, full shave-down, sedated groom) is determined through a face-to-face or phone conversation with the pet owner, who is asked by program staff to describe their pet's grooming needs and behavior. Occasionally, a photograph of the pet is shared to determine the extent of the grooming services needed and the appropriate length of time. The client's residence and the pet's grooming needs determine at which service location (Bronx or Brooklyn) and service setting (CVC or mobile event) they will receive services. Proof of address or income is not required.

### Study design

Study procedures were approved by Advarra IRB (Pro00058852). All clients, regardless of grooming service type, were offered the opportunity to participate in a survey at the CVCs or a mobile event. Eligibility for receiving services was determined prior to completing the study survey. ASPCA staff emphasized that participation was entirely voluntary and would not impact the services or quality of care provided. Clients who chose to participate in the study were able to choose whether they wanted to complete a Spanish- or English-language version of the survey. We partnered with a professional translation company (Language Line) to translate the English version of the survey to Spanish. In addition, bilingual staff who work within the CE program offered feedback on the survey translation and adjusted phrasing to ensure the appropriateness of the survey for local Spanish-speaking clients in New York City.

After a client consented to participate, program staff explained the purpose of the survey, made efforts to ensure privacy and confidentiality of the survey responses, and offered clarification or assistance in completing the survey as needed. For clients receiving services at the CVCs, the survey consisted of two parts: a pre-groom survey (12 questions) and a post-groom survey (six questions). The pre-groom survey was given to the pet owner during the appointment check-in process and assessed four content areas: pet and owner demographics; the pet owner's view of the role of grooming in relation to maintaining their pet's health; the owner's confidence performing basic grooming tasks; and barriers they experience to grooming pets. After the grooming service was completed, the post-service survey was provided to the owner. The post-survey included two content areas: confidence level as it pertained to nail trimming (for nail trim demonstration appointments only) and supplies and supports that would help with maintaining grooming at home. Participants completed the survey on paper and/or using an iPad, depending on their preference, resources, and/or staff availability. To promote confidentiality and comfort, all questions were optional, participants were assigned a unique identifier, and completed surveys (i.e., paper) could be placed directly in a locked ballot box following completion. Procedures for participants recruited at mobile grooming events were modified due to the fast-paced nature of these events, as it was not possible to administer a post-groom survey. For this reason, all participants at mobile events received one survey that included the same set and order of questions as the CVC surveys, except for survey items about post-appointment confidence, which was not assessed. Survey questions are provided in Tables 1–**3**.

**Table 1:** Demographic characteristics reported by pet owners.

**Variable**			**% (*n*)/M (*SD*)**
	Pet type	Dog	90.4 (151)
	(*n* = 167)	Cat	9.6 (16)
Pet characteristics	Pet age	6 months to 7 years	59.3 (99)
	(*n* = 165)	Over 7 years	22.8 (38)
		5 months or younger	13.2 (22)
		Unsure	3.6 (6)
	Primary dog breed (*n* = 141)	Mixed Breed	22.8 (38)
		Shih Tzu*	21.6 (36)
		Yorkshire Terrier*	13.2 (22)
		Maltese*	4.8 (8)
		Pit Bull	4.8 (8)
		Poodle*	4.8 (8)
		Pomeranian*	4.2 (7)
		Jack Russell Terrier	1.8 (3)
		Chihuahua	1.2 (2)
		Border Collie	0.6 (1)
		French Bulldog	0.6 (1)
		Miniature Pinscher	0.6 (1)
		Miniature Schnauzer	0.6 (1)
		Saint Bernard	0.6 (1)
		Unknown	2.4 (4)
Participant characteristics	Gender	Woman	67.1 (112)
	(*n* = 144)	Man	16.8 (28)
		Nonbinary	1.2 (2)
		Prefer to self-describe	1.2 (2)
	Race/ethnicity	Black/African American	44.9 (75)
	(*n* = 163)	Hispanic/Latina/Latino/Latinx	34.7 (58)
		Multiracial/Mixed Race	14.4 (24)
		First Nations/Indigenous/Native American	0.6 (1)
		Asian/Asian American	0.6 (1)
		White/Caucasian/European American	0.6 (1)
		Prefer to self-describe	1.8 (3)
	Total household income	$0–25,000	62.9 (105)
	(*n* = 153)	$25,001–50,000	15.0 (25)
		$50,001^+^	0 (0)
		Prefer not to answer	13.8 (23)

* Included in analysis of medium to long-haired dog breeds.

### Participants

One hundred sixty-seven clients whose pet(s) received grooming services between December 21, 2021, and April 22, 2022, participated in the study. Nineteen clients (11%) received appointments for two or more pets. Clients who received services for multiple pets at the same appointment completed one survey. For clients who received services for multiple pets at the same appointment, one pet was randomly selected for inclusion in our analysis of pet demographic characteristics. Clients with multiple pets served at different times completed surveys at each appointment; however, we only used the first survey completed by the client in our analysis. Participants predominately identified as women (67%), Black or African American (45%), and reported a total household income under $25 K (63%). Ninety-two percent of participants were the primary caregiver of the pet served. Pets receiving services were predominately dogs (90%), between the age of 6 months and 7 years (60%), and of mixed breed (38%). Additional demographic characteristics of the participants and their pets are provided in Table 1. Clients were seen at two service locations (Bronx and Brooklyn) and three service settings: the Bronx CVC (22%), Brooklyn CVC (50%), and mobile events (28%). Among those who received services at a mobile event, 48% received services within the Bronx borough and 52% received services in Brooklyn.

### Measures

#### Grooming history and owners' perspective on the importance of grooming

Participants' prior efforts to groom their pet were assessed *via* two questions: (*1) Pet grooming includes activities like bathing your pet, trimming their nails, and brushing or trimming their hair. Has your pet ever been groomed by a professional before?* and (2) *Have you or a member of your family ever tried to groom your pet at home? If yes, what did you do? Please check all that apply*. Response options included: Bathe, trim nails, brush/comb hair, trim/cut hair, and “I have never tried to groom my pet at home.” Pet owners' view on the importance of pet grooming was assessed *via* one question measured on a 5-point scale (0 = not at all, 5 = very important). Specifically, the question read, “*How important do you feel regular grooming (brushing, bathing, trimming nails) is in keeping your pet healthy?*

#### Confidence performing basic grooming activities

Participants' confidence performing basic grooming activities was measured *via* four questions using a 10-point rating scale (1 = not confident at all, 10 = extremely confident). Specifically, we assessed confidence bathing, trimming nails, brushing/combing hair, and trimming/cutting hair. The prompt read, “*On a scale of 1 to 10, how confident are you in doing the following grooming activities for your pet at home?”*

#### Barriers to grooming

Barriers to grooming were assessed *via* the following question: “*Below are some reasons people can't groom their pets. Which of the following are true for you? Please check all that apply*.” The list of response options included 21 barriers (see Table 2), inclusive of an open-ended option (other reason). Participants could also endorse “*No issues–I am able to keep up with my pet's grooming needs at home or with a groomer.”* Each item was scored dichotomously (0 = not endorsed, 1 = endorsed). The total number of barriers experienced was calculated by creating a sum score comprised of each item and could range from zero to 21.

**Table 2:** Barriers to grooming pets.

**Below are some reasons people can't groom their pets. Which of the following are true for you? Please check all that apply**	**% (*n*)**
I do not have enough money to pay for grooming	56 (93)
I am scared that I might hurt my pet	45 (75)
I do not have enough money to buy grooming supplies (e.g., scissors, nail clippers, etc.)	29 (48)
Transporting my pet to the groomer is difficult.	17 (28)
I do not have a carrier to get my pet to the groomer	15 (25)
I am scared that my pet might hurt me.	15 (25)
I do not have someone to help me groom my pet.	13 (22)
My pet does not like to be groomed.	12 (20)
There are no groomers where I live	12 (20)
There are no pet supply stores close to where I live to buy grooming supplies (e.g., scissors, nail clippers, etc.)	11 (18)
I am scared the groomer will hurt my pet.	10 (17)
Grooming business hours do not line up with my schedule	9 (15)
I do not have time to take my pet to a groomer	8 (14)
I do not have time to groom my pet at home	8(13)
I am not physically able to control my pet.	7 (12)
My pet is not up to date on vaccines	7 (12)
I have no place to bathe my pet.	6 (10)
I am scared my pet will hurt the groomer.	4 (7)
Other	5 (9)
I do not have a leash to get my pet to the groomer	2 (4)
I do not have running water	0.01 (1)
No issues—I am able to keep up with my pet's grooming needs at home or with a groomer.	13 (22)

#### Supplies/supports needed

To identify ways to support pet owners in maintaining grooming at home, we asked, “*What supplies or supports would help you continue grooming your pet at home? Please check all that apply.”* Nine response options were provided (see Table 3), including an open-ended option (other). Participants also had the option of endorsing that they did not need anything. Each item was scored dichotomously (0 = not endorsed, 1 = endorsed). The total number of supplies/supports needed was calculated by creating a sum score comprised of each item and could range from zero to nine.

**Table 3:** Supplies and supports that would help pet owners maintain grooming at home.

**What supplies or supports would help you continue grooming your pet at home?**		**% (*n*)**
Brush/comb		61(102)
Nail trimmer		54 (90)
Hair clippers		38 (63)
Scissors		38 (63)
Behavioral support with my pet (obedience training class, dog trainer)		28 (47)
A reminder when it's time to groom my pet		23 (39)
Someone to help me		23 (38)
Access to reliable water or electricity		2 (3)
Other	2% (4)	Training in how to groom
		Shampoo
		Es aggresivo para cortar al pelo/He is aggressive for hair cutting
Don't need anything	9% (15)

#### Participant demographic questions

Demographic questions inquired about participants' gender, race/ethnicity, and income.

Gender was assessed *via* the following question: “*Of the following options, which feels most aligned with your gender?”* Response options included: man, woman, non-binary/genderqueer/agender, and prefer to self-describe. Race/ethnicity was assessed *via* the question: “*How do you identify your race and/or ethnicity? Please check all that apply.”* Nine response options were provided, including prefer to self-describe (open-ended). Income was assessed *via* one question: “*What is your approximate annual household income?” Seven* response options were provided reflecting $25 K increments, up to >$150,000. Participants could also choose “prefer not to answer.” Service setting (the Bronx CVC, Brooklyn CVC, mobile event) was identified by program staff by matching the participants' appointment date with the grooming program's service schedule.

### Analysis

All analyses were conducted in SPSS version 28.0. Participants' view on the role of grooming in maintaining their pet's health, prior grooming experiences, confidence with basic grooming tasks, and endorsement rates for individual barriers to grooming and supplies/supports needed were examined using basic descriptive statistics as appropriate. We conducted a series of Pearson chi-square tests of proportion to examine associations between our constructs of interest [individual barriers to grooming and supplies/supports needed, household income, service location (Brooklyn vs. the Bronx), and service setting (mobile event vs. CVCs)]. All test assumptions were checked prior to proceeding with analysis. When the chi-square test was not suitable (e.g., expected values in any of the cells of the contingency table < 5), a Fisher's exact test was used. When a statistically significant chi-square test was found (*p* < 0.05), we conducted *post-hoc* analyses of the standardized adjusted residuals for each cell in each contingency table to interpret the significant difference (10). Critical thresholds for these analyses were Bonferroni corrected to reduce the risk of Type I error. We conducted a series of independent sample *t*-tests to determine whether the average number of barriers to grooming and supplies/supports needed was significantly different across income groupings, service settings, and service locations. For all analyses pertaining to income, those who selected “prefer not to answer” were removed from the analysis.

## Results

### View on grooming, history of grooming, and confidence with basic grooming tasks

Eighty-nine percent of participants endorsed that they felt regular grooming (brushing, bathing, trimming nails) was very important (68%) or important (21%) in relation to efforts to keep their pet healthy. Eighty-three percent of the sample reported having attempted at least some form of grooming at home. Among those who had groomed pets at home, 70% had bathed their pet, 56% had brushed their pet, 40% had cut their pet's hair, and 20% had cut their pet's nails. Sixty-four percent of participants indicated their pet had been to a professional groomer, 31% had not, and 3% were unsure. Only 5% of the sample indicated their pet had not been groomed at home or by a professional groomer; 56% of these animals were 5 months of age or younger. On average, participants rated their confidence bathing their pet as seven on a 10-point scale (*SD* = ± 3.34). Similarly, the average confidence score for brushing hair was eight on a 10-point scale (*SD* = 3.10). Lower ratings were found for cutting nails, with an average score of three (*SD* = ± 2.90). Owners of medium- and long-haired breeds (*n* = 46) rated their confidence cutting hair as four on average (*SD* = ± 3.44); short-haired breeds were excluded from analysis due to the lack of relevance of this item to maintaining their pet's health. Exploratory independent samples *t*-tests were conducted to examine if pets' coat length was associated with owners' confidence performing basic grooming activities. No significant differences were found between owners of short-hair and medium- to long-haired breeds when comparing confidence bathing, brushing, and cutting nails (coat/hair-length is noted in Table 1).

### Barriers to providing basic grooming care

Barriers to grooming pets and rates of endorsement are provided in Table 2. Ninety-two percent of the sample experienced at least one barrier to grooming their pet. The median number of barriers experienced by participants was two (28% of the sample) and scores ranged from 0 to 17. Forty-six percent of the sample experienced three or more barriers to grooming their pet.

Exploratory chi-square analyses were conducted to examine associations between total household income categories and each barrier to grooming pets. Results indicated that individuals making < $25K were more likely than expected to report that they did not have enough money to pay for grooming, $\chi_{\left(1\right)}^{2}$ = 4.42, *p* = 0.04; specifically, 66.7% of participants with less than $25K total household income were unable to pay for grooming (adjusted residual = 2.1) compared to 44% of participants with total household incomes between $25,001 and $50K (adjusted residual = −2.1). No other significant associations were found between individual barriers to grooming and income. Results of an independent sample *t*-test indicated that participants who reported a total household income under $25K experienced significantly more barriers to grooming pets on average (*Mean* ± *SD* = 4.04 ± 3.29) compared to people with a total household income in the $25,001–50K range (*Mean* ± *SD* = 2.81 ± 2.15), [*t*_(142)_ = 2.41, *p* = 0.01].

We also examined whether the barriers participants faced to grooming and total number of barriers differed across service location. Results indicated that individuals served in Brooklyn were more likely than expected to report that they had no place to bathe their pet; specifically, 9% of participants served in Brooklyn endorsed this barrier (expected = 5%; adjusted residual = 2.4) compared to zero (adjusted residual = −1.4) participants served in the Bronx. Because more than one cell count was < 5, Fisher's exact test was examined, which indicated statistical significance of this association (*p* = 0.02). No other significant associations were found between service location and individual barriers to grooming. Results of an independent sample *t*-test indicated no significant difference in the mean number of barriers to grooming reported when comparing residents of the Bronx and Brooklyn, [*t*_(165)_ = 1.18, *p* = 0.24).

We also examined whether the barriers participants faced to grooming and total number of barriers differed across the service setting (brick-and-mortar CVCs vs. mobile). Three significant associations were found when examining the relationship between service setting and barriers to grooming. Results of a chi-square test of association indicated that pet owners who received grooming services at a CVC setting were more likely than expected to endorse that they did not have money for grooming supplies. Thirty-three percent of pet owners served at these settings endorsed this barrier (adjusted residual = 2.0), compared to 17% of people serviced through mobile grooming settings (adjusted residual = −2.0), $\chi_{\left(1\right)}^{2}$ = 3.99, *p* = 0.046. Similarly, those served through brick-and-mortar CVC settings were more likely than expected to report that they had no one to help groom their pet; specifically, 17% of participants served in CVCs endorsed this barrier (adjusted residual = 2.1) compared to 4% of participants served *via* mobile grooming events (adjusted residual = −2.1), $\chi_{\left(1\right)}^{2}$ = 4.32, *p* = 0.04. Owners of pets served at CVCs were also more likely than expected to indicate they were afraid they would harm their pet, $\chi_{\left(1\right)}^{2}$ = 7.11, *p* = 0.008. Approximately 51% of pet owners served at CVCs endorsed this barrier (adjusted residual = 2.7) vs. 28% of pet owners served *via* mobile grooming events (adjusted residual = −2.7). Results of an independent sample *t*-test indicated that participants who received services at CVCs reported significantly more barriers to grooming pets on average (*Mean* ± *SD* = 3.27 ± *SD* = 2.62) compared to people who received services through a mobile grooming event (*Mean* ± *SD* = 1.97 ± 1.60), [*t*_(132.33)_ = 3.86, *p* < 0.001].

### Supplies/supports needed

Endorsement rates for each type of supply/support needed are listed in Table 3. Ninety-one percent of the sample endorsed that at least one supply or support would help them maintain grooming at home. Brushes/combs were most frequently endorsed, followed by nail trimmers, hair clippers, scissors, behavioral support, reminders, someone to help, and access to reliable water/electricity. Among those who endorsed that a supply or support would help them maintain grooming at home, the number of supplies/supports needed ranged from 1 to 8, with a median score of three. More than half of the sample (52%) endorsed that three or more supplies/supports would assist them in maintaining their pet's grooming at home.

Exploratory chi-square analyses were conducted to examine associations between household income categories and each supply/support needed. No significant associations were found. Similarly, results of an independent samples *t*-test indicated there was not a significant difference in the total number of supplies/supports needed when comparing participants who reported a total household income under $25K and those who had a total household income in the $25,001–50K range. Chi-square tests of association were used to examine associations between service location and each type of support/service needed; associations between service setting and each support/service were also examined. No significant associations were found. In addition, results of an independent sample *t*-test indicated that the mean level of supports/services needed did not differ across service location [*t*_(165)_ = −0.45, *p* = 0.654] and service setting [*t*_(165)_ = 0.23, *p* = 0.819].

### Nail trimming demonstration

Thirty-five clients received tailored nail trimming demonstrations from CE's Grooming Specialist and completed surveys at pre- and post-demonstration. On average, clients rated their confidence trimming their pet's nails as three on a 10-point scale (*SD* = ± 2.12) pre-appointment and five out of 10 (*SD* = ± 2.91) on the post-appointment survey. Results of a paired-sample *t*-test indicated a statistically significant increase in clients' confidence trimming nails following the nail trimming demonstration, *t*_(34)_ = −2.61, *p* = 0.003. The range of change in confidence from pre-demonstration to post-demonstration was −4 to 8, with 68% of participants indicating higher scores post-demonstration, 22% demonstrating no change, and 10% reporting lower levels of confidence post-demonstration. Exploratory analyses were conducted to examine potential demographic differences across these groups; we found no significant associations with pet or pet owner characteristics and/or service location or setting.

## Discussion

This study addresses a gap in research on animal welfare and pet owners' access to grooming-related services and supplies by examining factors that serve as obstacles and facilitators of maintaining pets' grooming needs in a sample of subsidized pet grooming service recipients. Our findings indicate that most (95%) pet owners who were served by the grooming program and chose to participate in the survey had groomed their pets at home and/or had brought their pet to a professional groomer previously. In addition, a majority of participants indicated that they felt grooming was important or very important in maintaining their pet's overall health. Few animals served by the program had not received some form of grooming-related care in their lifetime and most pets who had not been groomed were < 5 months of age. Vaccine and wellness visits for young animals are important times to educate pet owners regarding their animals' needs as they grow, and it is essential that pets' grooming needs are part of the client-DVM dialogue in the same way vaccine and other preventative care is seen as part of the preventative care package. Furthermore, these early visits are an opportunity to strengthen owner-veterinarian relationships and support owner-pet attachment. Prior studies indicate that these relationships increase owner compliance with veterinary recommendations and overall care of the pet (11). Thus, it is important for veterinary professionals to include pets' grooming needs in these early discussions with pet owners and follow up on these discussions in subsequent visits.

Most pet owners in the current study (89%) endorsed that grooming is important or very important to their pet's health. Our findings mirror results of Rohlf et al.'s online survey of Australian dog owners which identified that ~79% of owners groomed their dog regularly and most (83%) agreed that grooming was necessary (75%) and good for a dog's health (77%) (8). Although most participants in the current study acknowledged the importance of grooming, 92% reported experiencing at least one barrier to maintaining their pet's grooming and nearly half (46%) experienced three or more barriers to providing grooming. In addition, 91% endorsed that at least one supply/support would be beneficial in maintaining their pet's grooming needs at home and more than half reported that three or more supplies/services would be beneficial. Collectively, these findings support our hypotheses that most participants would report experiencing multiple barriers to providing grooming care and benefit from multiple supplies/supports. Furthermore, these findings suggest that the ASPCA's grooming program is reaching a key group of pet owners who understand the connection between maintaining grooming and their pet's overall health but would benefit from continued services that reduce barriers to providing this type of care, such as low- or no-cost service and supply provision. This demonstrates the important role of subsidized grooming services as a potential means of preventing grooming-related omissions of care and reducing animal suffering.

All 21 hypothesized barriers to care assessed in our study were endorsed by at least one participant. However, only one barrier to maintaining pets' grooming—not being able to afford pet grooming—was endorsed by more than half the sample. Finances were also a barrier to purchasing grooming supplies for 29% of the sample. This is not surprising given the total household income criteria for community members' eligibility to receive services through this subsidized program. Still, our findings further underscore the critical relationship between individual and family finances and pet owners' access to health-related services and supplies for companion animals. Although lack of access to veterinary care is a problem that has been an increasingly prevalent topic in research and discussion, particularly as it pertains to pets and people living in poverty, access to grooming services (including vaccinations to qualify for grooming services) and supplies have typically been omitted from these conversations (4, 11). Given important links between grooming-related care and animals' health and wellbeing, our findings support prior arguments that access to grooming services and supplies should be included in dialogue, programming, and research on animals' access to health-related services (4).

Among barriers to grooming pets at home, fear of hurting the pet was endorsed by 45% of the sample, indicating a potential need for resources such as hands-on training, video tutorials, and text- and image-based guides that can provide long-term aid to the owner. Animal welfare organizations and veterinary professionals can address this need by providing educational resources that are readily available online and at their brick-and-mortar locations, as well as empowering staff to proactively talk to clients about grooming their pets and field any questions and concerns, particularly from new pet owners. The combination of verbal discussions with written support materials has been demonstrated in other topic areas (i.e., human medicine) to be an effective way to promote owner comprehension and retention of care information (12). For example, Kessels and Roy (12) examined empirical evidence concerning obstacles to remembering medical information and models on effective communication in human medical practice. Synthesizing findings across diverse areas of medicine, they concluded that individuals' ability to recall or remember medical information is often poor and marked by inaccuracies, particularly among anxious and/or older individuals. They emphasize that instructions for treatment are particularly difficult to recall and therefore, they recommend the use of simple and specific instructions and supplementing verbal instructions with written or visual material. They conclude that visual communication aids are especially effective in low-literacy individuals. Few studies on adherence and/or compliance (consistency and accuracy with which a patient follows a prescribed regimen) to a mode of treatment have been published in the veterinary medicine literature (13). We are unaware of any studies concerning recommendations for pet grooming. Identifying the cause(s) of mismatches between veterinarian or groomer recommendations and pet owners' actions could be valuable for recognizing opportunities to improve veterinarian- or groomer-client interactions and the quality of animal care (12). As noted by Abood (13), challenges to adherence include economic concerns, issues of convenience, time constraints, and the ability to convince owners of the benefit of a recommendation. This mirrors the current study's findings regarding barriers to grooming pets (13).

Sharing knowledge about alternative methods and tools for trimming nails [e.g., nail files, grinders, Qwik-stop (a powder that controls minor bleeding caused by trimming nails)] and making these resources available may also be helpful. Further, it may be worthwhile for veterinary practices, animal welfare programs, and humane law enforcement officers to develop an overall grooming/health guide for dogs and cats that incorporates nail care tips along with guidance on grooming activities (e.g., bathing, trimming hair), supply recommendations, and suggested YouTube, Instagram or TikTok videos to view; such a guide could be made available and reviewed with the client during service appointments. Veterinary practices could include these resources in new pet packages that are commonly provided to clients during initial wellness visits. In addition, shelters and rescues could provide such resources to residents in their community and at the time of adoption, with an option for pet owners to opt in to receiving reminders (text, email, mail) about their pet's grooming needs and/or integrate questions about pet grooming into post-adoption surveys. When creating resources such as a grooming guide, it is essential to ensure these resources are available in languages and formats that are accessible to pet owners from diverse backgrounds. These resources may be particularly beneficial for individuals adopting medium and long-haired breeds. Although owners of those breeds did not report more concerns or barriers in the current study, prior research shows that these dogs and cats may be particularly vulnerable to grooming-related omissions of care (1, 4). Other potential models of providing resources include the development of web and mobile applications, such as DogMate, which was recently developed to serve current and prospective dog owners in the Philippines. Available online and offline, this web and mobile application showcases general information on dog breads and their specific health and hygiene needs with attention to the country and environmental context; grooming instructions based on coat-type and related illustrations are also provided (14).

Animal welfare organizations, veterinary clinics, and humane law enforcement professionals could also work collaboratively with community members to develop a system to share community resources. “Libraries of Things” have become increasingly popular in recent years and offer individuals and non-profit agencies no- or low-cost access to an inventory of tools to complete various projects. For example, “tool libraries” (which often stock and lend out various tools for home improvement) are common and many argue that they cut waste, save money, and contribute to community sustainability by lowering economic barriers to home improvement (15– 17)^2^. It may be beneficial to work with existing Libraries of Things and their staff to ensure existing programs are equipped with animal care tools (e.g., hair clippers) and supplies (e.g., carriers) that serve as barriers to grooming and ensure that local pet owners are informed about the availability of these resources. In addition, it may be beneficial to create and promote a library specific to pet care supplies and supports, whereby staff are able to maintain grooming tools and members are able to consistently access appropriate grooming supplies that are in good condition (e.g., nail trimmers, filers) as well as other supplies (e.g., shampoo, tubs, carriers, and leashes) when needed. Variability of the species served, and the care characteristics required to maintain different types of hair should be considered, as well as the need for referrals or additional resources for skin problems or other medical conditions (e.g., parasites) that cannot be addressed by such programs. This model of community-sharing may be particularly beneficial in underserved communities where there is a dearth of groomers and pet supply stores and/or for pet owners who require assistance to groom their pet, as was evident among 23% of our sample. Alternately and/or additionally, animal control officers or similar professionals could conduct supportive conversations with pet owners to help improve their ability to perform grooming related tasks. These professionals could have grooming tools available and offer similar resources to pet owners during home visits, thereby addressing barriers to transportation, a barrier to grooming that impacted 17% of our sample. If they are not able to offer these services on the spot, they could partner with an allied organization or service provider that the pet owner could be referred to using a simple referral card. Future research should investigate whether such models are effective in reducing barriers to maintaining pet health.

In the current study, we found that the total number of barriers to grooming differed by income groups and service setting, with pet owners making under $25K and receiving services in a CVC setting facing more barriers to grooming. Regarding income, those with income under $25K were more likely to report that they did not have enough money to pay for grooming. Indeed, 86% of people who endorsed this barrier had incomes below $25K. This suggests that, if faced with limited resources, grooming programs in these areas could consider prioritizing program eligibility to families making under $25K. In addition, those who received services at the CVC setting experienced more barriers to grooming on average and were more likely than expected to endorse not having money for grooming supplies, not having someone to help groom their pet, and being afraid they would hurt their pet. Income, location (borough), and service setting were not statistically significantly associated in the current study; therefore, more research is needed to understand why CVC clients appear to be more at risk for not having help with grooming, not being able to afford supplies, being fearful of harming their pet, and experiencing more barriers to grooming pets. This finding may stem from the fact that sedated grooming can only take place where veterinarian observation can be provided, which was available at the CVCs but not mobile settings. Sedated grooming is required when the pet's behavioral, physical and/or haircoat condition would interfere with regular grooming because it would be unsafe for the pet or persons or cause undue stress or pain for the pet to be groomed without sedation. For this reason, pets seen at CVCs may have had relatively more severe grooming needs that were influenced by the greater number of barriers to grooming experienced by their owners. Future research is needed to explore these hypotheses.

An interesting and unexpected finding is that some residents of Brooklyn (albeit a small proportion) reported not having any place to bathe their pets whereas no residents of the Bronx reported this barrier. It is unclear whether this can be attributed to random variation or to some meaningful difference between boroughs—and if the latter, what the nature of that difference might be. Further, not having a place to bathe one's pet could mean anything from not having a suitable sink/tub, to not wanting or being allowed to use the sink/tub for bathing the pet, to not having housing, among other interpretations. Future research should continue to examine the extent to which clients report this as a barrier and more directly assess the underlying reasons, with the aim of informing potential solutions. This finding also relates to the planned acquisition of a pet. Not only should potential pet owners consider factors related to the animal when identifying a pet that is a good match—environmental factors should be considered as well to support humane and compassionate care.

Given that most participants were relatively confident in their ability to bathe and brush their pets at home, our findings suggest that it may be particularly important for animal welfare programs and services that aim to improve access to pets' health care *via* direct services to focus on provision of nail trimming services and demonstrations, since owners may be able to maintain other aspects of grooming on their own when equipped with appropriate supplies and supports (e.g., hair clipper, reminders). Since nail-trimming may be one of the most stressful steps when grooming a pet, habituation, or desensitization, to this process at an early age is a key reason for pet owners to perform nail-trimming without hurting the animal or themselves. Our findings indicate that nail trimming demonstrations may be a cost-effective way to provide longer-term, sustainable support to pet owners who will ultimately need to provide this basic level of care throughout the pet's years of life. Results of this study help clarify that dedicated time and staff that provide nail trimming demonstrations may be a worthwhile investment with important implications for preventing grooming-related omissions of care. It would be helpful for future research to explore whether an increase in pet owners' confidence following a nail trimming demonstration is associated with their ability to maintain nail-trimming at home and/or their confidence when performing the task independently at home. Although our study suggests that brief nail trimming demonstrations are successful in achieving statistically significant increases in pet owners' perceived confidence trimming nails, on average, participants rated their post-appointment confidence at the mid-point (“5”) on our scale, indicating the need for further support and skill-building. In addition, it is important to highlight that nearly a third of participants who received nail-trimming demonstrations did not report increased confidence following the service (with some reporting lower levels of confidence). More research is needed to understand how, why, and for whom these demonstrations are/are not effective and how they can be improved to serve all clients effectively and efficiently. In addition, it may be beneficial to test whether this type of intervention can be effectively scaled up to provide demonstrations to small or large groups *via* in-person methods and/or *via* virtual platforms (e.g., Zoom). Integrating assessments of animal stress and welfare may also be beneficial to establish interventions that employ best methods for promoting animal welfare while being maximally beneficial to pet owners (9, 18, 19).

Collectively, our findings regarding barriers to grooming and supplies and supports needed to maintain grooming suggest that a one-time grooming service and/or demonstration is just one aspect of a spectrum of services needed to prevent grooming-related omissions of care. It is critically important that programs that aim to improve access to animal health services consider ways to ensure that clients have supports/supplies to maintain grooming at home. Although the need for basic grooming supply items (e.g., brushes) was endorsed most frequently in our sample, a notable 30% indicated that behavioral support services would assist them in maintaining their pet's grooming at home. Behavior concerns can be a barrier to maintaining grooming because some pets may become fearful, anxious, and/or stressed during grooming related tasks and, consequently, may exhibit aggressive and potentially dangerous behaviors (to self and person) (9, 20). It is likely that negative grooming-related experiences may cause pet owners to be stressed and fearful of attempting grooming again, potentially putting the pet at risk of their grooming needs being neglected and contributing to negative impacts on the human-animal bond (4, 21). Seven percent of participants in the current study said they could not physically control their pet, 13% said they had no one to help them groom their pet and as previously mentioned, 23% said that having someone to help them groom their pet would assist them in maintaining their pet's grooming at home. Supports and trainings that help pet owners foster positive animal behaviors during grooming [e.g., *Cooperative Care* (22), *Fear Free*^®^^3^] may assist owners who face such barriers. Research is needed to understand links between pet owners' negative grooming experiences and future grooming-related care, along with prevention/intervention approaches that can assist in ameliorating this issue.

### Limitations and future directions

Although our study has many strengths and addresses a notable gap in the literature, there are several limitations that warrant consideration. First, this study used cross-sectional convenience sampling, which impacts the generalizability of our results. Improving access to grooming services, facilitating access to grooming supplies, and improving caregivers' knowledge of their pets' grooming needs are all likely to improve the welfare of a significant number of companion animals. However, it is important to consider that all participants in the current study were clients of subsidized grooming services. Future research is needed to understand barriers to grooming and supplies/supports needed in other subgroups of pet owners (e.g., those not receiving subsidized services, other income brackets, geographic regions, etc.). Another limitation concerns the fast-paced nature of the grooming services, which prevented us from tracking how many clients declined participation in the study or determining how they may differ from those who consented to taking the survey. People who chose to participate in the study may have held more positive views of pet grooming and/or may have been motivated to participate based on their strong need for grooming-related resources. Future studies would do well to employ probability sampling in order to increase generalizability of results. It may be advantageous to randomly assign participants to a “demonstration-only” vs. “demonstration plus resource guide” group, or other combinations of interventions and resources to identify what service or combination of services and supports is most effective in assisting pet owners with maintaining grooming at home. Relatedly, although we made notable efforts to ensure confidentiality of survey responses, social desirability response bias may have contributed to participants choosing a higher rating of their confidence following the nail trimming demonstration.

It is also important to note that the grooming program did not accept cats at mobile grooming events for the first few months of the program, thus potentially impacting the representation of felines in the study. Furthermore, the coat length of dogs and cats who received grooming services was not explicitly assessed. Thus, cats were omitted from our analysis of medium- and long-hair breeds since breed was not assessed, and we were only able to include dogs in our analysis if their owner endorsed one breed type and we could confidently assume they had medium- to long-hair [which omitted dogs whose owner selected “mixed breed” from consideration and those of breed origins with multiple coat types (e.g., smooth vs. long coat Chihuahua)]. Future research would benefit from examining cats and dogs separately, to identify whether there are similar and/or unique barriers to grooming across species or different supports and services needed to maintain grooming at home. Similarly, understanding differences across dog breed size would be beneficial, particularly in relation to travel and behavioral challenges. Future studies would also benefit from asking participants who had multiple pets receiving services at the same time to provide unique responses for each pet, which was not possible in the current study due to staff capacity.

Another limitation pertains to the readability, relevance, and comprehensiveness of survey items in relation to our sample. Although this study employed several processes and procedures to ensure the survey was easily understood and implemented, qualitative research with clients served by the program, and other members of the community, would help ensure that all barriers and supports relevant to grooming are assessed in future research and that the language used to describe barriers and supports is clear and easily understood. It is also possible that some aspects of the Spanish-language survey may not have been appropriate for all Spanish-speaking clients due to linguistic variations across cultures and countries of origin. For example, one client selected “other” supply/support was needed to maintain their pet's grooming and wrote, “Es aggresivo para cortar al pelo” (He is aggressive for hair-cutting) but did not select that behavioral support for the pet or someone to help would assist them in maintaining their pet's grooming. This may be because the client did not view behavioral training as a means to reduce aggressive behavior and/or that our terminology was confusing or lacked cultural or linguistic relevance. Relatedly, the Bronx and Brooklyn are culturally, racially, and ethnically diverse areas, with a notable proportion of residents who speak languages other than English and Spanish. Thus, results of this study are not inclusive of these minority language speakers (e.g., Chinese, Russian, Kru, Ibo, Yoruba).

Due to the nature of grooming appointments and staff capacity, we were also unable to examine how long it had been since the pets served by the program had been groomed and how often participants provided basic forms of pet grooming. Thus, our study is also limited by not knowing the context and frequency of participants' pet grooming activities, and our methodology does not allow us to delineate differences in attitudes toward grooming and barriers/supports needed among pet owners who are regularly able to meet their pet's grooming needs vs. those who are not. Understanding barriers associated with chronic or long-term inability to meet a pet's grooming needs would be helpful in preventing serious animal welfare issues involving grooming-related omissions of care. It would also be beneficial for future studies to ask about participants' perception of how often pets require grooming. Another limitation concerns our inability to examine differences in barriers to grooming in relation to pet owner age. Although our survey inquired about participants' age, a notable proportion listed their pet's age instead of their own, causing us to exclude age data from analyses. Older adult pet owners may face more barriers to grooming pets due to a greater likelihood of mobility issues and/or disabilities and may benefit from approaches tailored to those barriers (4, 23). We also did not account for family size or number of pets, which may have implications for how income level impacts the association between income and barriers to grooming.

## Conclusion

The current study examined factors and services that operate as barriers and facilitators of pet grooming among clients served by a subsidized grooming service program in New York City. Study results suggest that income and cost of services/supplies serve as primary barriers to grooming pets, along with several other factors including fear of hurting the pet, transportation, and pets' behavioral issues. Our findings suggest that a brief, tailored nail trimming demonstration may be an effective service that animal welfare organizations, veterinary clinics, and humane law enforcement can provide to increase pet owners' confidence in performing this grooming task at home. However, our findings also suggest that a one-time grooming service and/or demonstration is just one aspect of a spectrum of services needed to prevent grooming-related omissions of care. It is important that low- and no-cost grooming programs that focus on underserved communities ensure that clients also have supports/supplies needed to continue to maintain grooming at home and that stakeholders in animal welfare (e.g., veterinarians, animal shelters and rescue organizations) work collaboratively with community members to reduce barriers to pet owners' access to these services. Further examination and identification of factors (e.g., how the service is delivered, pet owners' individual and situational characteristics) that contribute to lasting impacts of nail trimming demonstrations and other grooming services on pet owners' maintenance of pet grooming may help identify specific methods and subsets of clients who benefit the most from these services. We recommend several directions for future research, including longitudinal research to understand the impact of subsidized grooming services and supply provision on pet owners' ability to maintain their pet's grooming needs over time.

## Data availability statement

The raw data supporting the conclusions of this article may be made available by the authors upon request.

## Ethics statement

This study was approved by Advarra IRB Pro00058852. The participants provided written informed consent to participate in this study.

## Author contributions

SM, CD, and JS: conceptualization and methodology. SM and AM: analysis. SM, JS, LK, LN, MG, and CD: writing, review, and editing. All authors contributed to the article and approved the submitted version.

## Funding

This study was funded and conducted by the ASPCA.

## Conflict of interest

The authors declare that the research was conducted in the absence of any commercial or financial relationships that could be construed as a potential conflict of interest.

## Publisher's note

All claims expressed in this article are solely those of the authors and do not necessarily represent those of their affiliated organizations, or those of the publisher, the editors and the reviewers. Any product that may be evaluated in this article, or claim that may be made by its manufacturer, is not guaranteed or endorsed by the publisher.

## Footnotes

^1^Mobile grooming is a service that typically involves a pet groomer who travels to pet owners' homes and/or a specific community location and performs grooming services from specially outfitted vehicles.

^2^ https://www.theguardian.com/society/2019/apr/24/library-of-things-borrowing-scheme-conquer-world

^3^ https://www.fearfreehappyhomes.com/content-library/?training-grooming
